# The divergent effects of strong NHC donation in catalysis†
†Electronic supplementary information (ESI) available: Rate profiles for decomposition of ***u*-GIIm** and ***s*-GIIm**; X-ray crystallographic details; NOESY spectra, and derivation of the [PCy_3_]-independence of decomposition. CCDC 1400077. For ESI and crystallographic data in CIF or other electronic format see DOI: 10.1039/c5sc02592c


**DOI:** 10.1039/c5sc02592c

**Published:** 2015-10-06

**Authors:** Justin A. M. Lummiss, Carolyn S. Higman, Devon L. Fyson, Robert McDonald, Deryn E. Fogg

**Affiliations:** a Center for Catalysis Research & Innovation and Department of Chemistry , University of Ottawa , Ottawa , K1N 6N5 , Canada . Email: dfogg@uottawa.ca; b Department of Chemistry , University of Alberta , Edmonton , T6G 2G2 , AB , Canada

## Abstract

Strong σ-donation from NHC ligands (NHC = N-heterocyclic carbene) is shown to have profoundly conflicting consequences for the reactivity of transition-metal catalysts. Such donation is regarded as central to high catalyst activity in many contexts, of which the second-generation Grubbs metathesis catalysts (RuCl_2_(NHC)(PCy_3_)(=CHPh), **GII**) offer an early, prominent example. Less widely recognized is the dramatically inhibiting impact of NHC ligation on initiation of **GII**, and on re-entry into the catalytic cycle from the resting-state methylidene species RuCl_2_(NHC)(PCy_3_)(=CH_2_), **GIIm**. Both **GII** and the methylidene complexes are activated by dissociation of PCy_3_. The impact of NHC donicity on the rate of PCy_3_ loss is explored in a comparison of ***s*-GIIm**, *vs.****u*-GIIm**, in which the NHC ligand is saturated H_2_IMes or unsaturated IMes, respectively. PCy_3_ loss is nearly an order of magnitude slower for the IMes derivative (a difference that is replicated, albeit smaller, for the benzylidene precatalysts **GII**). Proposed as an overlooked contributor to these rate differences is an increase in the Ru–PCy_3_ bond strength arising from π-back-donation onto the phosphine ligand. Strong σ-donation from the IMes ligand, coupled with the inability of this unsaturated NHC to participate in significant π-backbonding, amplifies Ru → PCy_3_ π-back-donation. The resulting increase in Ru–P bond strength greatly inhibits entry into the active cycle. For ***s*-GII**, in contrast, the greater π-acceptor capacity of the NHC ligand enables competing Ru → H_2_IMes back-donation (as confirmed by NOE experiments, which reveal restricted rotation about the Ru–NHC bond for H_2_IMes, but not IMes). Ru → PCy_3_ back-donation is thus attenuated in the H_2_IMes complexes, accounting for the greater lability of the PCy_3_ ligand in ***s*-GIIm** and ***s*-GII**. Similarly inhibited initiation is predicted for other metal–NHC catalysts in which a π-acceptor ligand L must be dissociated to permit substrate binding. Conversely, enhanced reactivity can be expected where such L ligands are pure σ-donors. These effects are expected to be particularly dramatic where the NHC ligand has minimal π-acceptor capacity (as in the unsaturated Arduengo carbenes), and in geometries that maximize NHC–M–L orbital interactions.

## Abstract 2

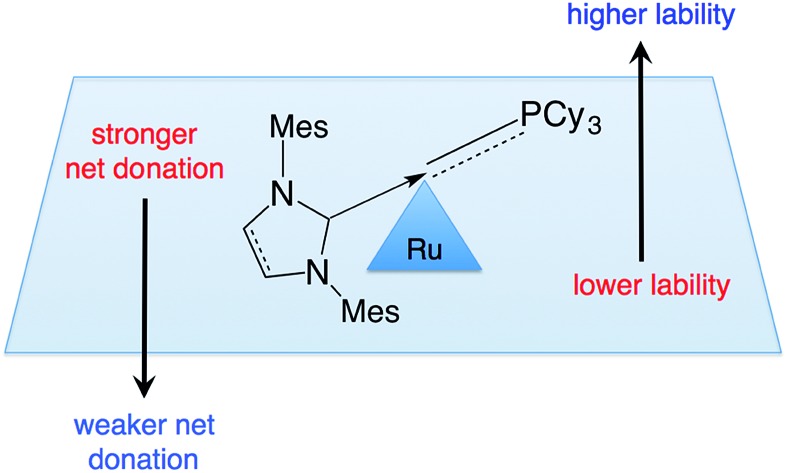
The inverse relationship between NHC donicity and catalyst initiation.

## Introduction

The remarkable impact of N-heterocyclic carbene (NHC) ligands on transition-metal catalysis^1^–^4^ is due largely to their strong σ-donor character, a feature highlighted in even the earliest reviews.^5^–^7^ Strong NHC binding is believed to inhibit decomposition of molecular catalysts,^1^,^8^ and to stabilize the higher oxidation states essential in multiple catalytic contexts, including olefin metathesis and cross-coupling reactions.^1^–^3^ As well, however, emerging work points toward the potential for NHC donation to influence bonding interactions with other ligands present, both ancillary ligands and bound substrate.^9^–^11^

In a leading recent example, the Neidig group reported evidence for ground-state weakening of the Fe–Cl bond by σ-donation from the NHC ligand in tetrahedral FeX_2_(NHC)_2_ complexes.^9^ The implied potential labilization of π-donor ligands by NHC ligands is of keen interest. The potentially broad implications of such behaviour in catalysis prompted us to explore the impact of NHC donicity on neutral, dative donor ligands, particularly in geometries that reinforce inter-ligand electronic communication. Here we demonstrate the impact of the NHC ligand on *trans*-ligand binding, in an important example drawn from olefin metathesis.

**Fig. 1:**
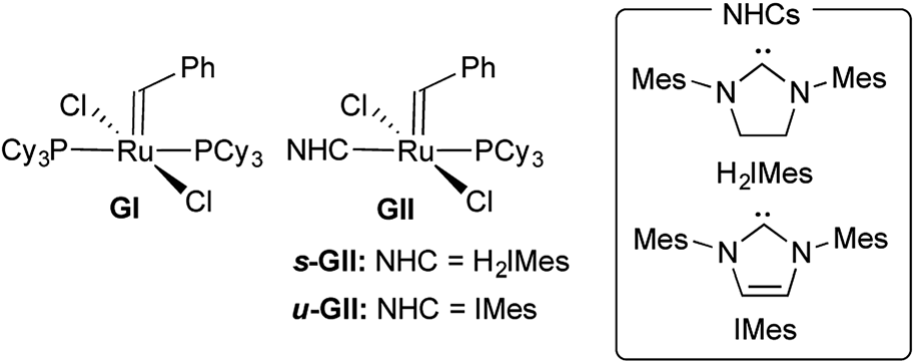
The first and second-generation Grubbs catalysts, **GI** and **GII**.

The breakthrough activity of the second-generation Grubbs catalysts,^12^,^13^ which greatly expanded the scope of the reaction relative to the parent system **GI** (Fig. 1), was originally attributed to labilization of the σ-donor PCy_3_ ligand by the strongly donating *trans*-NHC ligand.^14^ In a seminal kinetics study, however, Grubbs and co-workers demonstrated that PCy_3_ loss is in fact slower for **GII** than the first-generation catalyst **GI**.^14^

A leading explanation for this “inverse *trans* effect” highlights alkylidene rotation as a trigger for PCy_3_ dissociation, pointing out higher torsional barriers to such rotation in the NHC complexes.^15^ An alternative view emerges from Kennepohl's discovery, based on groundbreaking X-ray absorbance spectroscopy (XAS) studies, that the Ru center in ***s*-GII** is more electropositive than that in **GI**.^16^ This implies that the NHC ligand is a poor net charge donor, relative to PCy_3_. An increased electrostatic attraction between the more electron-deficient Ru center in **GII** and the strongly-donating PCy_3_ ligand was proposed to account for the reduced phosphine lability.

**Fig. 2:**
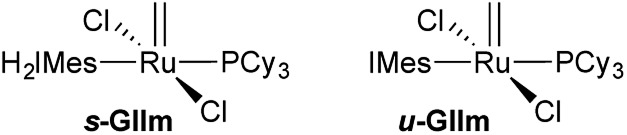
The off-cycle resting states for **GII**: methylidene complexes ***s*-GIIm** and ***u*-GIIm**.

Adopting the majority view of NHC ligands as strong σ-donors, we speculated that NHC donation might itself be a factor: that strong σ-donation could in fact strengthen the *trans* Ru–PCy_3_ bond, by increasing Ru → PCy_3_ backbonding. In exploring this possibility, we focused on the methylidene species **GIIm** (Fig. 2), to eliminate steric or π-stacking effects associated with the benzylidene moiety, and electronic perturbation arising from benzylidene π-acidity. **GIIm** is, moreover, a key player in catalysis, as the resting-state species in most ring-closing and cross-metathesis reactions promoted by **GII**. That is, because **GIIm** is thermodynamically stable relative to both the benzylidene precatalyst **GII**, and other ruthenium species present in the catalytic cycle, its concentration builds up during metathesis. Recently-developed^17^ routes to the second-generation methylidene complexes enable their direct study.

The availability of the closely related complexes ***u*-GIIm** and ***s*-GIIm** permits the effect of NHC donicity on *trans*-PCy_3_ bonding to be assessed with minimal extraneous perturbation.^18^,^19^ The π-acceptor capacity of saturated NHCs such as H_2_IMes, first proposed more than a decade ago, has seen much discussion.^10^,^11^,^16^,^19^–^29^ In recent years, the focus has shifted to means of deconvoluting NHC σ-donor and π-acceptor properties.^23^–^26^ While unsaturated Arduengo NHCs are generally viewed as poor π-acceptors, accumulating evidence suggests that their saturated analogues can exhibit significant π-acidity.^10^,^11^,^16^,^19^–^28^ If σ-donation from the H_2_IMes ligand in ***s*-GIIm** is countered by Ru–NHC backbonding, we considered that this should result in experimentally observable distinctions between the H_2_IMes and IMes complexes, which could potentially be correlated with differences in PCy_3_ lability.

Here we quantify the differences in PCy_3_ lability in **GIIm**; we demonstrate that strong σ-donation from the H_2_IMes ligand is indeed tempered by π-backbonding onto the NHC, as evidenced by restricted rotation about the Ru–H_2_IMes bond, and that PCy_3_ loss is dramatically slower for the IMes system, in which NHC σ-donation is unrelieved by NHC π-acidity (as confirmed by room-temperature rotation about the Ru–IMes bond). Based on these observations, we propose that enhanced backbonding onto the PCy_3_ ligand is a key, overlooked contributor to the low phosphine lability characteristic of the second-generation Grubbs catalysts. Such Ru → PCy_3_ backbonding relieves the heightened electron density at Ru that would otherwise result from strong NHC σ-donation, and consequently strengthens the Ru–P bond. The broader implications for catalysis are discussed.

## Results and discussion

### Assaying PCy_3_ lability for **GIIm**

Direct assessment of PCy_3_ lability for the second-generation methylidene complexes is hampered by a combination of strong phosphine binding and thermal instability. Even for the more labile benzylidene pre-catalysts, PCy_3_ loss from the IMes derivative ***u*-GII** was 640 times slower than from the first-generation complex **GI**.^14^ Qualitative evidence indicated drastically lower lability for the methylidene complexes **GIIm**, but attempts to measure rate constants were thwarted by decomposition at the temperatures required to induce PCy_3_ exchange (*ca.* 85 °C).^14^

This underscores the point that the thermodynamic stability of **GIIm** relative to other catalytically relevant species does not equate to stability against decomposition. Indeed, the methylidene complexes are significantly more vulnerable than their benzylidene precursors, owing to their susceptibility to nucleophilic attack at the Ru=CH_2_ site.^30^–^32^

**Scheme 1:**
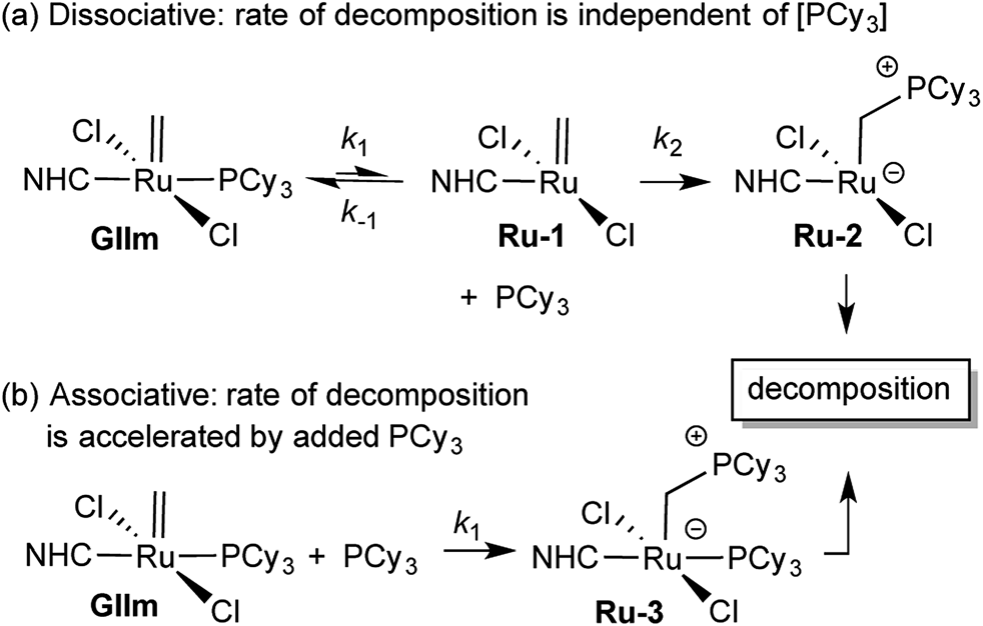
Predicted [PCy_3_] dependence for decomposition of **GIIm***via* associative and dissociative pathways.^33^ For rate law derivations, see the ESI.†

We considered that this vulnerability, which constituted a problem in the original exchange experiments, could offer a disguised opportunity to assess phosphine lability. Specifically, if decomposition of **GIIm** proceeds *via* rate-limiting loss of PCy_3_,^30^ then the rate of decomposition reports on the rate of PCy_3_ loss. To confirm that this reaction proceeds only *via* four-coordinate **Ru-1**, we examined the impact of added PCy_3_ on the reaction kinetics. If phosphine attack occurs on **Ru-1** (Scheme 1a), the rate of decomposition should be unaffected, for the reasons discussed below. If, however, **GIIm** can react directly with PCy_3_ (Scheme 1b), decomposition should be accelerated.

**Fig. 3:**
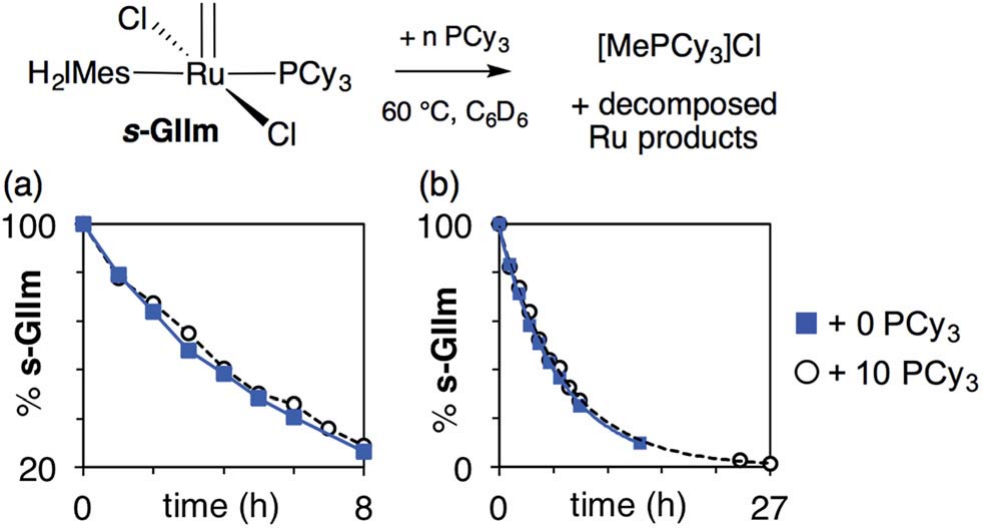
Assessing the rate of decomposition of ***s*-GIIm** in the presence and absence of added PCy_3_. (a) Over the first 8 h. (b) Over the full period of decomposition.

As seen from Fig. 3, the rate of decomposition is unaffected by added PCy_3_, indicating reaction *via* the dissociative pathway (Scheme 1a). The preference is unsurprising, given steric restrictions on the approach of PCy_3_ to the methylidene carbon in five-coordinate **GIIm**. The absence of an inverse dependence on [PCy_3_] may at first seem inconsistent with rate-determining loss of PCy_3_. This reflects the participation of PCy_3_ in the *k*_2_ step (*i.e.* the **Ru-1** → **Ru-2** transformation), as well as the *k*_–1_ step (the **Ru-1** → **GIIm** back-reaction). If nucleophilic attack on **Ru-1** is much faster than phosphine re-binding (*i.e. k*_2_ ≫ *k*_–1_), the rate expression reduces to *k*_1_ [**GIIm**] (see ESI†).

(For completeness, it may be noted that even if *k*_2_ and *k*_–1_ were of comparable magnitude – or indeed if *k*_2_ ≪ *k*_–1_ – no phosphine inhibition would result. Because the rate of the *k*_–1_ step is *k*_–1_ [**Ru-1**][PCy_3_], and that of the *k*_2_ step is *k*_2_[**Ru-1**][PCy_3_], any change in [PCy_3_] alters both rates equivalently, and the phosphine concentration term cancels out. Thus the rate of reaction is independent of [PCy_3_], irrespective of the relative magnitudes of *k*_2_ and *k*_–1_).

**Fig. 4:**
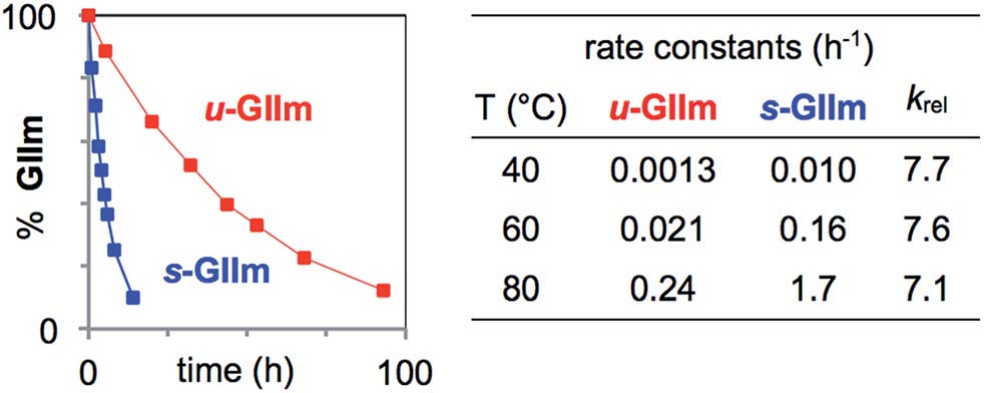
Assessing rates of PCy_3_ loss from the decomposition of ***s*-GIIm** and ***u*-GIIm** in C_6_D_6_. Left: Rate curves at 60 °C. Right: Initial rate constants and *k*_rel_ (normalized to ***u*-GIIm**) at 40 °C, 60 °C, and 80 °C. For half-lives and rate plots at other temperatures, see the ESI.†

To assess the rates of PCy_3_ loss from ***s*-GIIm** and ***u*-GIIm**, in the present case, where *k*_2_ ≫ *k*_–1_, we measured the rates of decomposition of these complexes in C_6_D_6_. Decreases in the proportion of **GIIm** over time were established by ^1^H NMR analysis. The integrated intensity of the methylidene singlet was measured relative to 1,3,5-trimethoxybenzene (TMB; *δ* C*H* 6.26 ppm) as internal standard. Decomposition was nearly eight times faster for ***s*-GIIm** than ***u*-GIIm**, as shown by the rate curves in Fig. 4. The relative rates show little change from 40–80 °C: in each case, loss of PCy_3_ from the IMes derivative was 7–8 times slower. DFT studies by the Jensen group reported an identical trend for the parent benzylidene catalysts, with *k*_1_ for ***u*-GII** being seven-fold lower than for ***s*-GII**.^34^

**Fig. 5:**
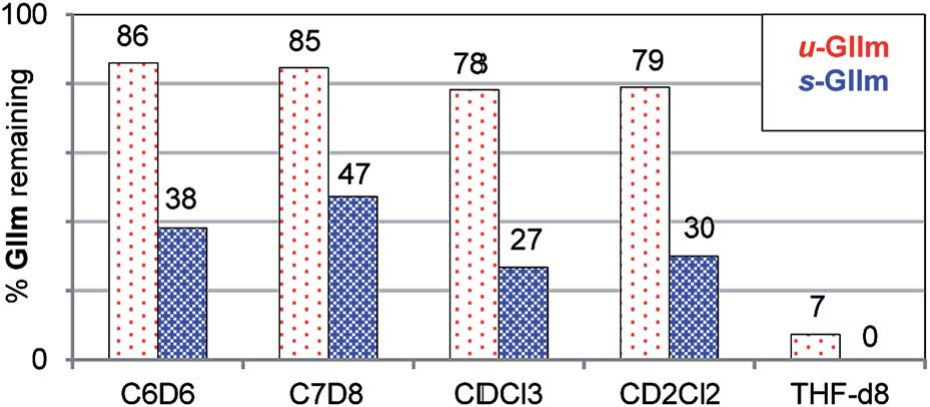
Assessing the relative stability of ***u*-GIIm** and ***s*-GIIm** in common solvents, as a proxy for PCy_3_ lability (6 h, 60 °C oil-bath; ^1^H NMR integration *vs.* TMB). Key chemical shift data for **GII** and **GIIm** in these solvents are tabulated in the ESI.†

The lower phosphine lability of ***u*-GIIm** relative to ***s*-GIIm** was maintained in other solvents (Fig. 5). In these experiments, the proportion of **GIIm** remaining after 6 h at 60 °C was measured. Decomposition was marginally faster in chlorinated media than in aromatic solvents, and dramatically faster in the coordinating solvent THF. The solvent-dependence of PCy_3_ dissociation thus follows the trend C_7_H_8_ ∼ C_6_H_6_ < CH_2_Cl_2_ ∼ CHCl_3_ ≪ THF, for both the IMes and H_2_IMes methylidene complexes. This agrees with the trend previously established for initiation of the benzylidene precatalyst ***s*-GII**, for which the rate-determining step is likewise PCy_3_ loss.^14^

The consistency in these reactivity patterns, as well as the excellent agreement with the relative rate constants computed by Jensen (see above), validate the use of decomposition rates to quantify rates of PCy_3_ loss from **GIIm**. Also noteworthy is the close correlation between relative rates of initiation of **GII** in different solvents, and relative rates of decomposition of **GIIm**. This correlation accounts for the observation that increasing the rate of initiation does not improve reaction rates for the Grubbs catalysts.^35^ Instead, because productive metathesis generates an unprotected methylidene moiety, faster initiation is offset by faster methylidene abstraction by free PCy_3_.

### Crystallographic analysis: comparison of ***u*-GIIm** with ***s*-GIIm**

**Fig. 6:**
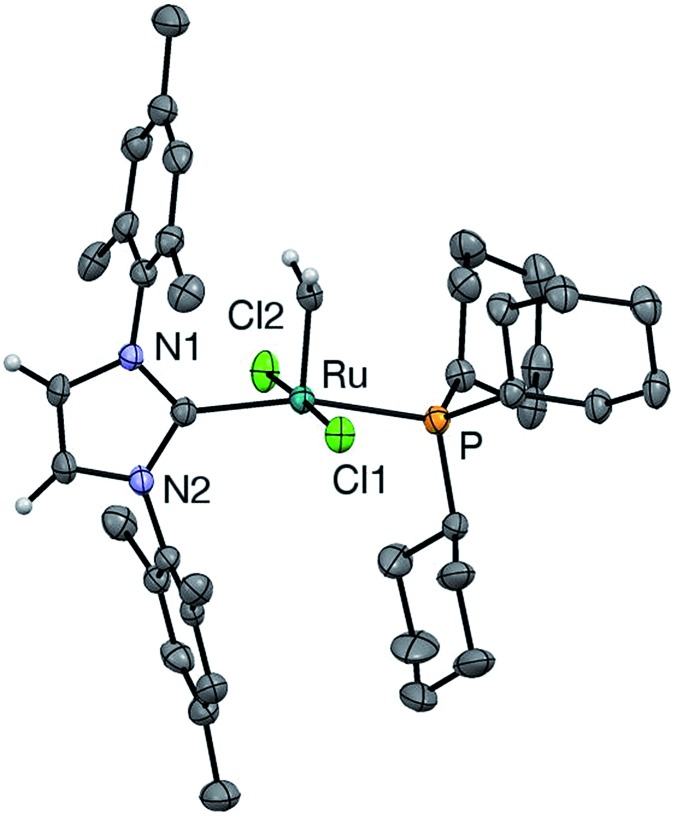
Perspective view of ***u*-GIIm**. Non-hydrogen atoms are represented by Gaussian ellipsoids at the 30% probability level. Hydrogen atoms on methylidene and NHC backbone carbons are shown with arbitrarily small thermal parameters; other hydrogens are not shown.

In the hope of gaining insight into the bonding interactions that distinguish the IMes and H_2_IMes analogues, we undertook a crystallographic study of ***u*-GIIm**, for comparison with the reported structure of ***s*-GIIm**.^36^ The instability of these complexes in solution can be minimized by low-temperature handling, and X-ray quality crystals of ***u*-GIIm** deposited from concentrated solutions in toluene over days at –35 °C. The ORTEP plot is shown in Fig. 6; key bond lengths and angles are compared with those for ***s*-GIIm** in Table 1.

**Table 1:** Key bond lengths and angles for **GIIm** complexes

	***u*-GIIm**	***s*-GIIm** 36
***τ*-parameter**	0.19	0.19

**Bond lengths (Å)**		
Ru–P	2.4174(16)	2.427(1)
Ru=C	1.797(7)	1.800(2)
Ru–C_NHC_	2.077(5)	2.065(2)
Ru–Cl(1)	2.389(2)	2.393(1)
Ru–Cl(2)	2.381(2)	2.379(1)

**Bond angles (°)**		
Cl–Ru–Cl	176.99(6)	177.05(2)
P–Ru–C_NHC_	165.63(16)	165.81(5)
P–Ru=C	97.2(2)	96.90(7)
Cl(1)–Ru=C	93.1(2)	92.89(7)
Cl(2)-Ru=C	89.9(2)	89.77(7)
C_NHC_–Ru=C	97.2(3)	97.29(8)

The geometry at Ru is square pyramidal in both cases, as indicated by the *τ* values of 0.19 (*cf. τ* = 0 for a perfect square pyramid, and *τ* = 1 for a perfect trigonal bipyramid).^37^ While the P–Ru–C_NHC_ angle shows some distortion from the 180° ideal (*ca.* 166° in both ***u*-GIIm** and ***s*-GIIm**), excellent orbital communication is expected between the *trans*-disposed phosphine and NHC ligands. Importantly, however, the Ru–P bond distances in ***s*-GIIm** and ***u*-GIIm** are statistically indistinguishable, despite the nearly tenfold difference in phosphine lability. The absence of a correlation between Ru–PCy_3_ bond length and bond strength was pointed out for the parent benzylidene complexes,^14^ but has gone widely unnoticed. Frenking has pointed out that metal–ligand bond lengths are not reliable indicators of bond strength, where the ligand can function as an acceptor as well as a donor.^38^ The π-acceptor properties of the phosphine ligand in the NHC complexes are discussed below.

### Molecular dynamics study: Ru=C_NHC_ rotation and bond order

**Fig. 7:**
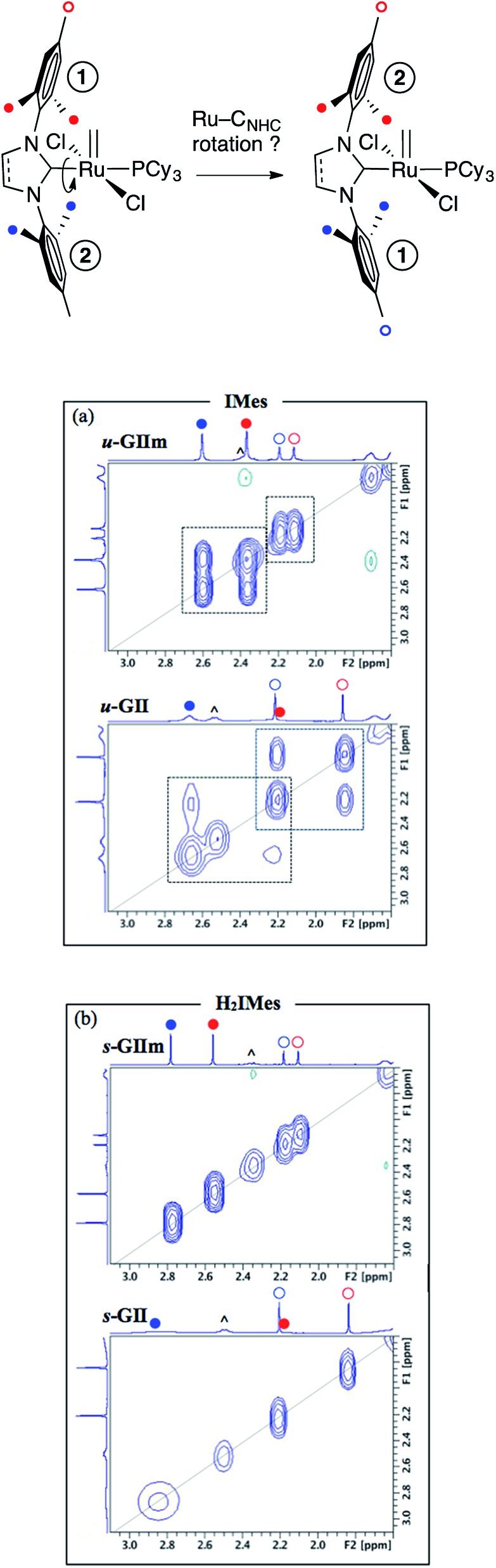
^1^H–^1^H NOESY spectra showing dependence of Ru–NHC rotation on NHC unsaturation. (a) Exchange correlations between mesityl methyl signals for the IMes derivatives. (b) Absence of correlations for the H_2_IMes analogues. (all in C_6_D_6_, 500.1 MHz, 25 °C, 1.5 s relaxation delay). Symbols: (^) = Cy; for others, see top.

More direct insight emerged from a molecular dynamics study, in which 2D NOESY-NMR was used to assess rotational exchange between the mesityl rings above and below the basal plane (Fig. 7, top). Exchange cross-peaks were observed for all four unique mesityl methyl signals in ***u*-GIIm** and ***u*-GII**, indicating rotation about the Ru–IMes bond at room temperature (Fig. 7a). No such cross-peaks were evident for ***s*-GIIm** and ***s*-GII** (Fig. 7b), even for the well-resolved *p*-Me singlets (the *o*-Me singlets are less well resolved, perhaps due to [Ru]=CHPh swiveling). Slower rotation of the H_2_IMes ligand in both the methylidene complex ***s*-GIIm** and its benzylidene parent ***s*-GII** is important in indicating that restricted rotation is unrelated to the steric demand of the [Ru]=CHR substituent.

Restricted rotation about the Ru–H_2_IMes bond implies increased Ru–C_NHC_ double-bond character, arising from π-back-donation from the metal onto the vacant *p*-orbital on the NHC carbon. Free rotation of the IMes ligand, in contrast, indicates a high proportion of single-bond character in the Ru–C_NHC_ bond. This accords with the experimental and computational findings described above, showing stronger π-acceptor character for the H_2_IMes ligand than IMes. Bertrand and co-workers drew a similar conclusion in a comparative study of H_2_IPr–PPh and IPr–PPh adducts, also on the basis of a solution dynamics study (IPr = 1,3-bis(2,6-diisopropylphenyl)imidazol-2-ylidene).^23^ Thus, the saturated H_2_IPr derivative was classified as a phosphaalkene species, and the unsaturated IPr adduct as a phosphinidene.

### Origin of the inverse *trans* effect

As noted in the Introduction, the origin of the dramatically reduced phosphine lability in the second-generation Grubbs catalysts is a puzzle of long standing. Straub suggested that faster PCy_3_ loss from **GI** is due to repulsive interactions between the chloride ligands and the β-hydrogen atoms of the cyclohexyl rings.^39^ More recently, Yang, Truhlar and co-workers reported DFT evidence showing that alkylidene rotation functions as a toggle to trigger PCy_3_ dissociation, and that the torsional barriers to rotation are higher for ***s*-GII**.^15^

Kennepohl's XAS study stands out, however, for the unexpected revelation that ***s*-GII** exhibits a higher 1s ionization potential for Ru – that is, a more electron-deficient metal center – than does the first-generation parent **GI**. We suggest that this is due to enhanced π-donation from Ru onto the NHC and PCy_3_ ligands. It should be noted that the Kennepohl study examined this possibility for ***s*-GIIm**. It was rejected, as calculations at the level of theory then available indicated limited Ru → PCy_3_ backbonding (in consequence of which, stronger PCy_3_ binding was attributed to an enhanced Ru/PCy_3_ electrostatic attraction). Importantly, however, consideration of dispersion forces has since emerged as critical to quantitative evaluation of the PCy_3_ dissociation step.^40^

The limited role heretofor assigned to Ru–PCy_3_ π-acceptor interactions in this system is perhaps unsurprising, given the widespread perception of alkylphosphines as strong σ-donors and weak π-acceptors (a situation also encountered in the context of NHC donicity; see above). Here too, however, a re-evaluation is in progress. In an analysis of electron density and structural effects, Leyssens, Harvey and co-workers demonstrated that π-backbonding from the metal atom onto the P–R σ*-antibonding orbitals can represent a significant component of metal–phosphine bonding, including for trialkylphosphine complexes.^41^ A recent leading review of computational approaches to the understanding of metal–phosphorus bonding likewise emphasizes that calculated ligand descriptors for phosphine ligands must consider their π-acceptor character.^42^

**Fig. 8:**
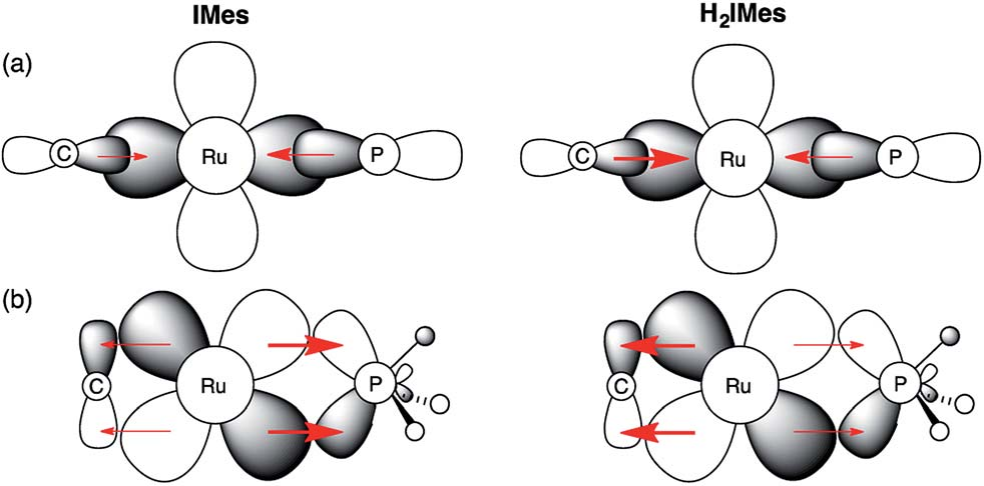
Impact of NHC π-acidity on PCy_3_ lability. (a) σ-Bonding interactions; (b) π–bonding interactions. Perspective down the Ru=CHR bond.

In light of these developments, we suggest that π-back-donation onto the phosphine is a significant, overlooked contribution to the low PCy_3_ lability in the second-generation Grubbs catalysts. The potent σ-donor properties of the NHC ligand constrain back-donation onto any π-acceptor ligands present. For precatalyst ***s*-GII**, three ligands can participate in π-backbonding: H_2_IMes, PCy_3_, and benzylidene.^39^ In the case of ***u*-GIIm**, the poor π-acceptor character of the IMes and methylidene ligands leaves the PCy_3_ ligand as the sole entity that can ameliorate the buildup of charge on the metal. We propose that this buildup is offset for ***u*-GIIm** by greater Ru → PCy_3_ back-donation (Fig. 8), and for ***s*-GIIm**, by greater Ru → H_2_IMes back-donation, accompanied by a lesser amount of Ru → PCy_3_ back-donation. This would account for the poor net charge donation from the saturated NHC ligand observed in the Kennepohl study. Also relevant in this context is an energy decomposition analysis by Poblet and co-workers, which suggested that the π-acceptor capacity of H_2_IMes reduces total charge donation to the metal for ***s*-GIIm**, relative to its IMes analogue.^21^

Several consequences can be envisaged, which have a profound impact on catalytic behaviour. Most obviously, stronger Ru–P backbonding would account for the reduced lability of the PCy_3_ ligand in the IMes complexes, relative to their H_2_IMes analogues. Slower loss of PCy_3_ would in turn account for the 7–8-fold longer lifetime shown above for ***u*-GIIm**, relative to ***s*-GIIm**. Because phosphine dissociation is required for entry into the active catalytic cycle, however, the advantage of longer lifetime is offset by slower initiation for the precatalyst ***u*-GII**, and slower re-entry for the resting-state species ***u*-GIIm**. This proposal clarifies the greatly enhanced initiation efficiency of phosphine-free, Hoveyda-class metathesis catalysts,^43^ in which the π-accepting PCy_3_ ligand is replaced by a π-donating ether ligand, as well as the high latency of the Cazin catalysts, in which a much more strongly π-acidic phosphite ligand is present.^44^

In the Neidig study cited in the Introduction,^9^ the NHC ligands were shown to significantly reduce the binding strength of a chloride ligand in tetrahedral Fe–NHC complexes. The *strengthening* of the *trans*-PCy_3_ bond observed herein is a striking further manifestation of the impact of NHC donicity on M–L binding. Beyond the specific context of olefin metathesis, similar inhibition of uptake into catalysis may be expected whenever a π-acceptor ligand must be released in order to bind substrate, particularly where this ligand is *trans* to an NHC. Such effects are enhanced for systems in which the strong σ-donor character of the NHC ligand is undiminished by NHC π-acceptor capacity, as illustrated here for the IMes system.

## Conclusions

Strong NHC donation is arguably the defining feature of the second-generation Grubbs catalysts, as the parameter that enables their high inherent reactivity. The foregoing reveals that such strong donation wears a Janus face. Enhancing the electron density at the metal center activates the Ru-olefin intermediate, and stabilizes the Ru(iv) metallacyclobutane intermediate. However, it also greatly amplifies Ru → PCy_3_ backbonding: Ru–P bond strengths are thereby increased, and loss of phosphine is severely inhibited. This inverse *trans* effect is manifested in retarded initiation of the benzylidene precatalysts **GII**, and very slow re-entry into the catalytic cycle from the resting-state methylidene complexes **GIIm**.

Notwithstanding the central importance of the Grubbs catalysts and their descendents in olefin metathesis, the implications are considerably broader. The transformative impact of NHC ligands on homogeneous catalysis has long been assigned to their capacity to enhance the electron density at the metal. The influence of NHC donicity on the ancillary ligands, however, is now beginning to be examined more closely. The findings above contribute to emerging understanding of the profound impact of NHC donicity on M–L binding, and hence on catalytic behaviour. Specifically, inhibited initiation is predicted to be a general feature for M–NHC catalysts in which a π-acidic ancillary ligand occupies a latent substrate binding site, particularly where such ligands are *trans* to the NHC. The potential for activation of a π-accepting substrate located in this site is an obvious corollory. These findings complement recent work highlighting the *labilizing* effect of the NHC ligand on π-donor ligands in tetrahedral iron complexes. Differences in NHC π-acceptor capacity can thus either mitigate or reinforce *trans*-type M–L bonding interactions, with major consequences for catalyst conscription and activity.

## Experimental

### General procedures

Reactions were carried out under N_2_ using standard glovebox techniques, at ambient temperature (RT; 25–27 °C, unless otherwise noted). Dry, oxygen-free toluene was obtained using a Glass Contour solvent purification system. All NMR solvents (Cambridge Isotopes) were stored under N_2_ over Linde 4 Å molecular sieves for at least 6 h prior to use. Dimethyl terephthalate (DMT, >99%), 1,3,5-trimethoxybenzene (TMB, >99%), used as internal integration standards to support quantification in ^1^H NMR experiments, were obtained from Sigma-Aldrich. The methylidene complexes ***u*-GIIm** and ***s*-GIIm** were prepared by literature methods.^17^,^45^ X-ray quality crystals of ***u*-GIIm** were grown from toluene at –35 °C over 48 h.

NMR spectra were recorded on Bruker Avance 300 and 500 spectrometers at 23 °C (unless otherwise noted), and referenced to the residual proton of the solvent. Signals are reported in ppm, relative to TMS (^1^H) or 85% H_3_PO_4_ (^31^P) at 0 ppm.

### Representative procedure for measuring decomposition rates

In the glovebox, a J. Young NMR tube was charged with **GIIm** (10 mg, 0.013 mmol), TMB (*ca.* 0.5 mg), and C_6_D_6_ (660 μL). The sample was removed from the glovebox and a ^1^H NMR spectrum was measured to establish the initial ratio of ***s*-GIIm** to TMB. The NMR tube was then transferred to a 40 °C oil bath (thermocouple-equipped; ±1.5 °C). The rate was determined by collecting ^1^H NMR spectra at regular intervals. Rate profiles for ***u*-GIIm** and ***s*-GIIm** at 40 °C and 80 °C are given in the ESI.† To examine the [PCy_3_]-dependence of decomposition, a corresponding experiment was carried out with ***s*-GIIm** (9.2 mg, 0.0127 mmol), TMB (*ca.* 0.5 mg), and PCy_3_ (35.7 mg, 0.127 mmol, 10 equiv.) in C_6_D_6_ (635 μL) at 60 °C. Time-points were taken at regular intervals until decomposition was complete.

### Exploring the impact of solvent on decomposition of **GIIm**

These experiments were carried out as above at a bath temperature of 60 °C, with NMR analysis at a single time-point (6 h). Thermolysis experiments in CD_2_Cl_2_ (b.p. 40 °C) were carried out in thick-walled J. Young NMR tubes.

## Supplementary Material

Supplementary information

Crystal structure data
