# Supplementary material for: The divergent effects of strong NHC donation in catalysis
Source: Chem Sci. 2015 Oct 6;6(12):6739–46. doi: 10.1039/c5sc02592c (PMC5947514; doi:10.1039/c5sc02592c)
Supplement: Supplementary file 1 [file SC-006-C5SC02592C-s001.pdf]

Electronic Supplementary Material (ESI) for Chemical Science.
This journal is © The Royal Society of Chemistry 2015

Supplementary Information for:

The Divergent Effects of Strong NHC Donation in Catalysis

Justin A. M. Lummiss, Carolyn S. Higman, Devon L. Fyson, Robert McDonald,† and Deryn E. Fogg*

Center for Catalysis Research & Innovation, Chemistry Department, University of Ottawa, Ottawa, ON, Canada, K1N 6N5

Table of Contents

1. NOE spectra for ***s*-GIIm**, ***u*-GIIm**, ***s*-GII**, and ***u*-GII** S2

2. Inducing rotation in H_2IMes complex ***s*-GIIm** at elevated temperatures S4

3. Derivation of the [PCy_3] dependence of **GIIm** decomposition S5

4. Rate profiles for thermolysis of ***s*-GIIm** and ***u*-GIIm** at 40 °C and 80 °C S6

5. Half-lives for ***s*-GIIm** and ***u*-GIIm** at 40 °C, 60 °C, and 80 °C in C_6D_6 S7

6. Summary of key NMR data for **GIIm** and **GII** in a range of solvents S7

7. Crystallographic details for ***u*-GIIm** S7

8. References S8

S1. NOESY spectra for *s*-GIIm, *u*-GIIm, *s*-GII, and *u*-GII

† X-ray Crystallography Laboratory, Department of Chemistry, University of Alberta, Edmonton, Alberta, Canada, T6G 2G2

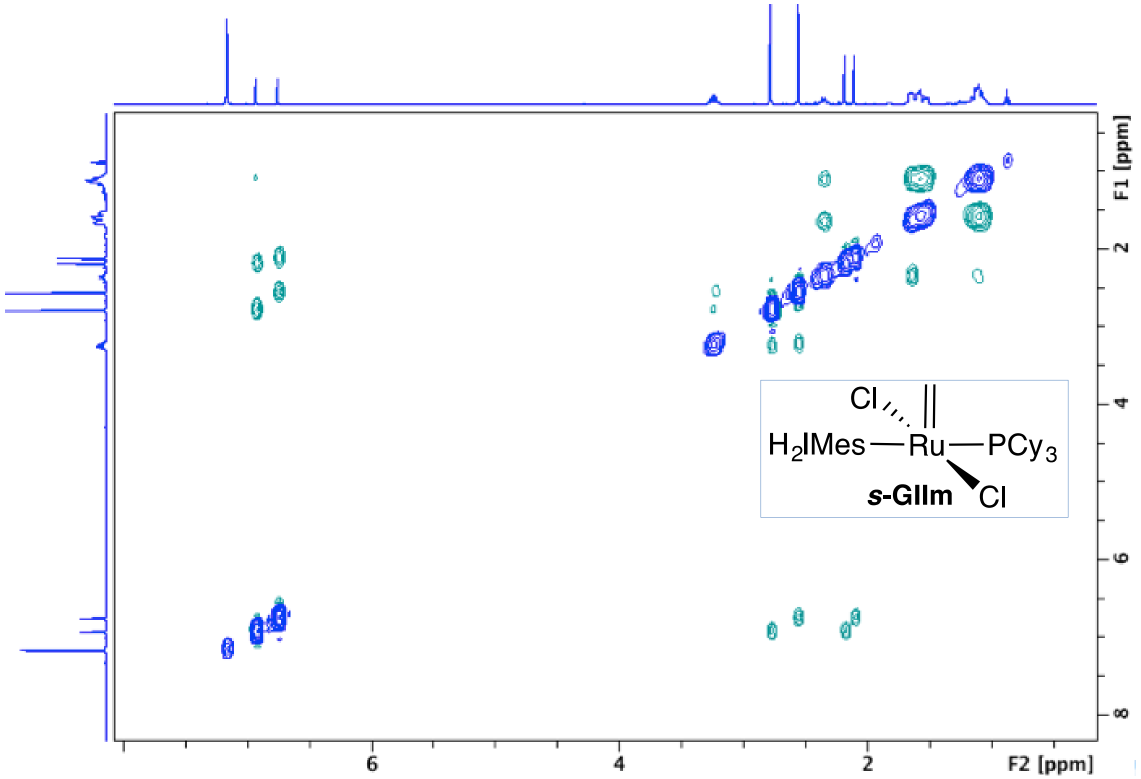

Figure S1. ^{1}H NMR NOESY spectrum (500.1 MHz, C_6D_6) for methylidene complex $RuCl_2(H_2IMes)(PCy_3)(=CH_2)$, ***s*-GIIm**.

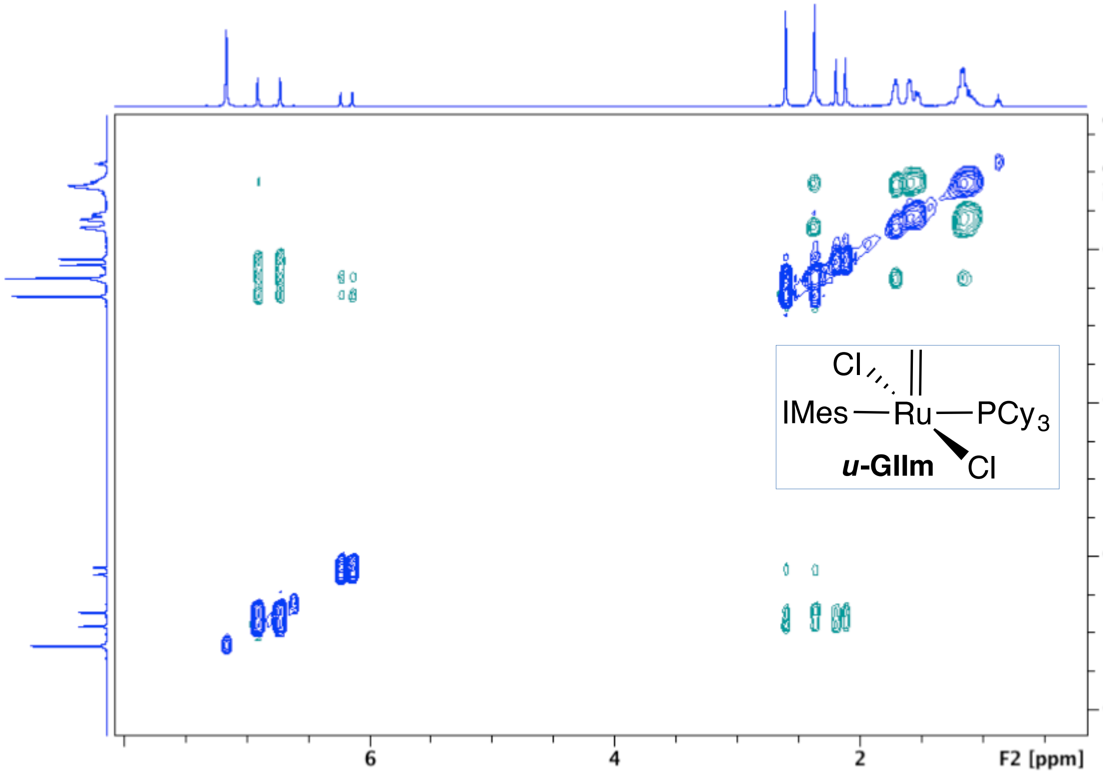

Figure S2. ^{1}H NMR NOESY spectrum (500.1 MHz, C_6D_6) for $RuCl_2(IMes)(PCy_3)(=CH_2)$, ***u*-GIIm**.

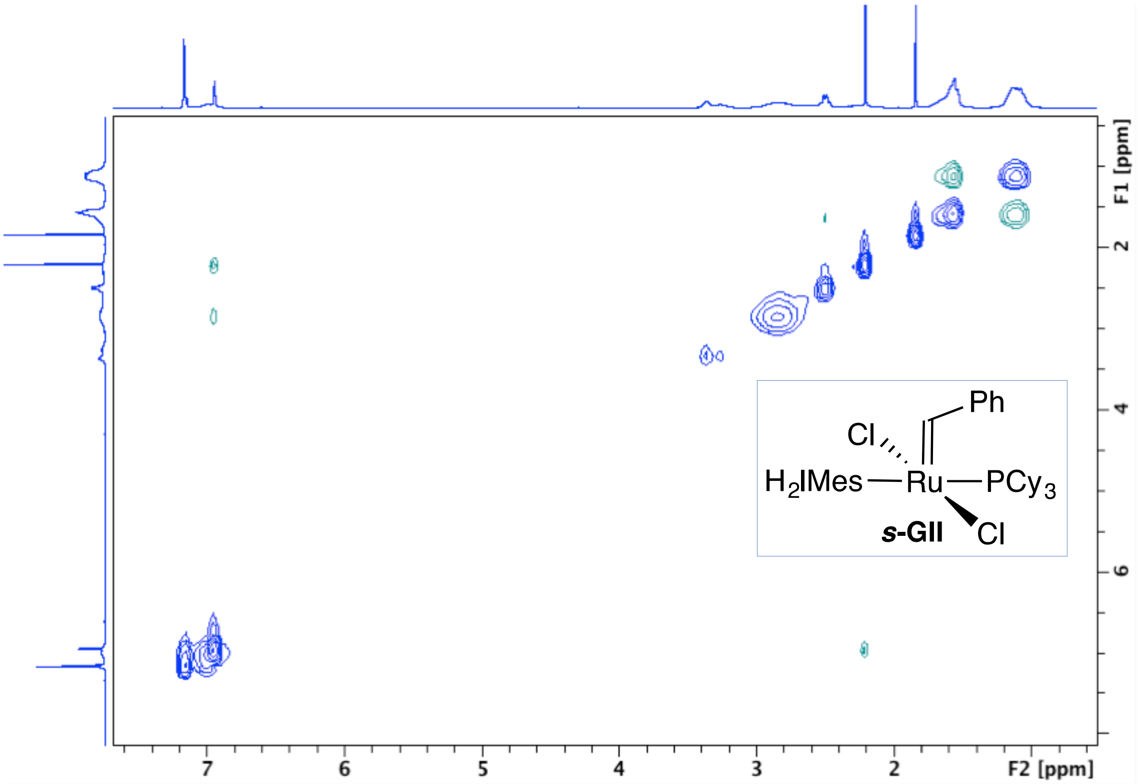

Figure S3. ^{1}H NMR NOESY spectrum (500.1 MHz, C_6D_6) for $RuCl_2(H_2IMes)(PCy_3)(=CHPh)$, ***s*-GII**.

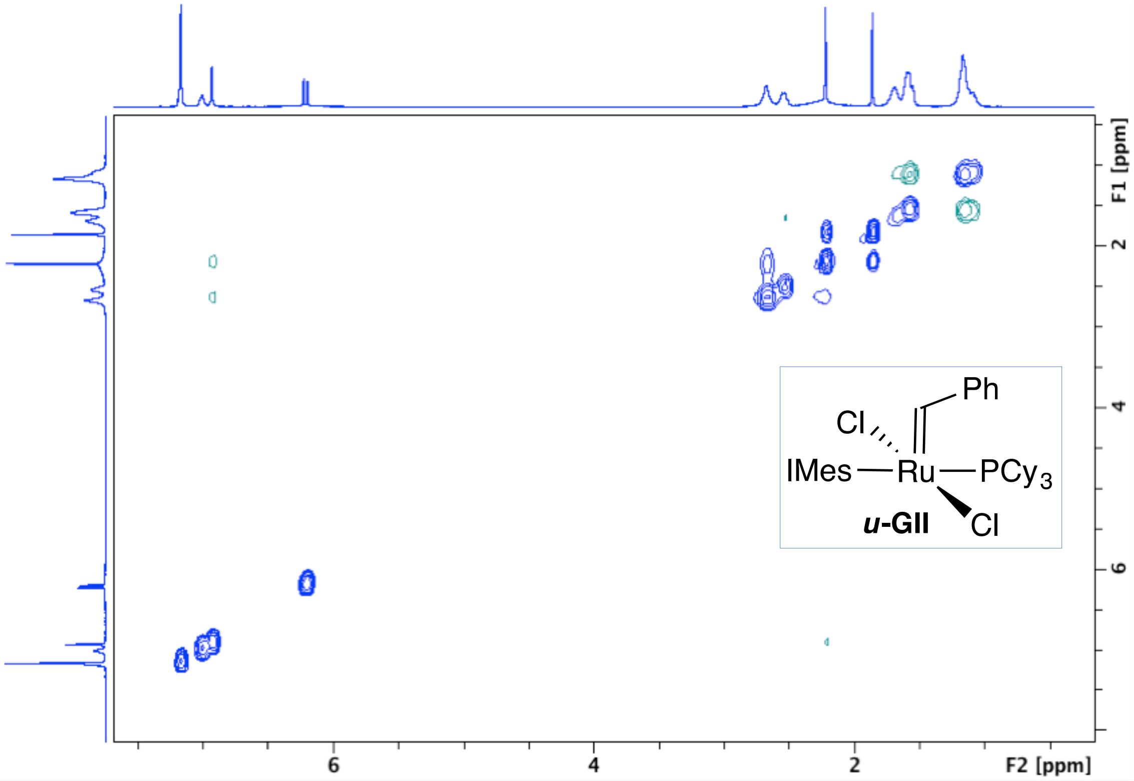

Figure S4. ^{1}H NMR NOESY spectrum (500.1 MHz, C_6D_6) for $RuCl_2(IMes)(PCy_3)(=CHPh)$, ***u*-GIIm**.

S2. Inducing rotation in *s*-GIIm by raising the temperature

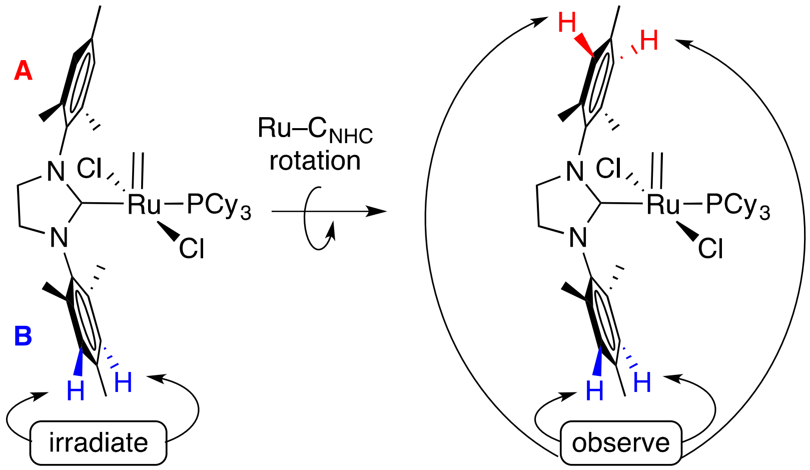

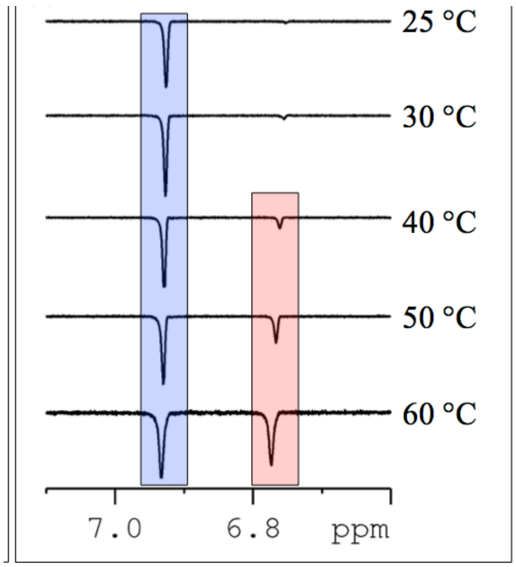

Figure S5. Inducing NHC rotation in ***s*-GIIm**. (a) Distinguishing mesityl rings by selective irradiation of the Mes C*H* signal for Ring **B**. (b) Onset of EXSY enhancement of C*H* signals in Ring **A** at 40 °C. ^{1}H NMR, 500 MHz, C_6D_6; 2 s mixing.

S3. Derivation of the $[PCy_3]$ dependence of GIIm decomposition

(a) Dissociative pathway

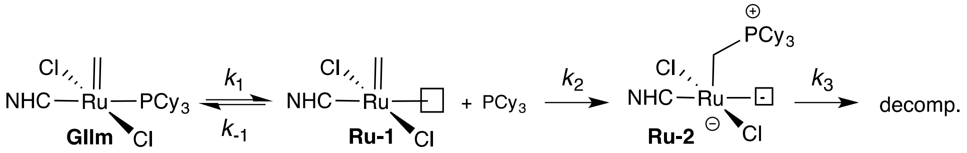

decomp. rate $= - d[\text{G}\mathbf{IIm}] / dt$

$= k_3 [\mathbf{Ru\text{-}2}]$

Applying the steady state approximation to spectroscopically unobservable **Ru-2**,

$$k_3 [\mathbf{Ru\text{-}2}] = k_2 [\mathbf{Ru\text{-}1}] [PCy_3]$$

$$[\mathbf{Ru\text{-}2}] = k_2 / k_3 \ [\mathbf{Ru\text{-}1}] [PCy_3]$$

$$\text{then rate} = k_2 [\mathbf{Ru\text{-}1}] [PCy_3]$$

Applying the steady state approximation to spectroscopically unobservable **Ru-1**,

$$k_1 [\mathbf{GIIm}] = k_{-1} [\mathbf{Ru\text{-}1}][PCy_3] + k_2 [\mathbf{Ru\text{-}1}][PCy_3]$$

$$[\mathbf{Ru\text{-}1}] = \frac{k_1 [\mathbf{GIIm}]}{(k_{-1} + k_2) [PCy_3]}$$

$$\text{then rate} = \frac{k_1 k_2 [\mathbf{GIIm}] [PCy_3]}{(k_{-1} + k_2) [PCy_3]}$$

$$= \frac{k_1 k_2}{(k_{-1} + k_2)} [\mathbf{GIIm}]$$

Thus the rate of decomposition is independent of $[PCy_3]$

(b) Associative pathway

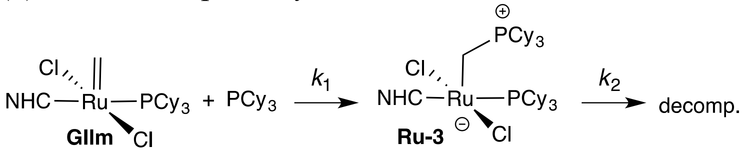

decomp. rate $= - d[\text{G}\mathbf{IIm}] / dt$

$= k_2 [\mathbf{Ru\text{-}3}]$

Applying the steady state approximation to spectroscopically unobservable **Ru-3**,

$$k_2 [\mathbf{Ru\text{-}3}] = k_1 [\mathbf{GIIm}] [PCy_3]$$

$$[\mathbf{Ru\text{-}3}] = k_1 / k_2 \ [\mathbf{GIIm}] [PCy_3]$$

$$\text{then rate} = k_1 [\mathbf{GIIm}] [PCy_3]$$

Thus the rate of decomposition has a first-order dependence on $[PCy_3]$

S4. Rate profiles for PCy_3 loss from *s*-GIIm and *u*-GIIm at 40 °C and 80 °C

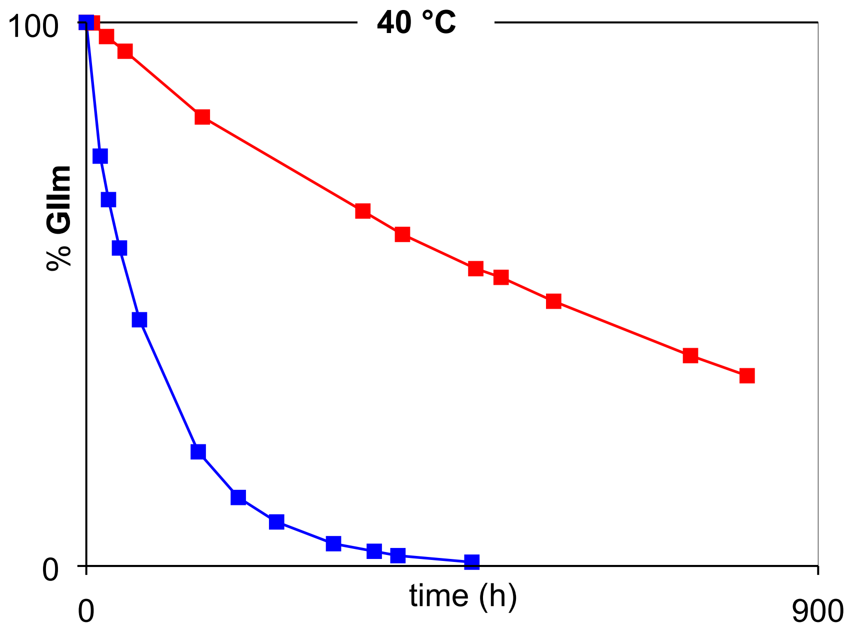

Figure S6. Assessing the stability of ***s*-GIIm** (blue) and ***u*-GIIm** (red) at 40 °C in C_6D_6 (^{1}H NMR integration vs. TMB as internal standard; measuring decreases in integration following t_0).

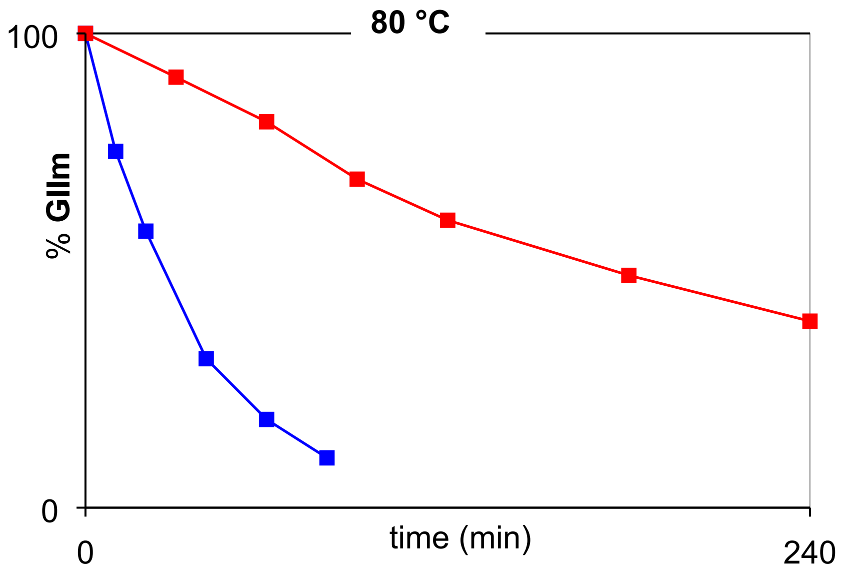

Figure S7. Assessing the stability of ***s*-GIIm** (blue) and ***u*-GIIm** (red) at 80 °C in C_6D_6 (^{1}H NMR integration vs. TMB as internal standard; measuring decreases in integration following t_0).

S5. Half-lives for *s*-GIIm and *u*-GIIm at 40 °C, 60 °C, and 80 °C in C_6D_6

Table S1. Half-lives data corresponding to the thermolysis experiments of Fig. 4.[a]

T (°C)	***u*-GIIm**	***s*-GIIm**
40	545	63
60	34	4.1
80	2.9	0.4

[a]Assessed by ^{1}H NMR: 300 MHz, TMB used as internal standard

S6. Key chemical shifts for GIIm and GII in common solvents

Table 2. Diagnostic ^{1}H and ^{31}P chemical shifts for **GIIm** and **GII** complexes.[a]

Solvent	δ_H (Ru=C*H*R)		δ_P (Ru-*P*Cy_3)	
R = H	***u*-GIIm**	***s*-GIIm**	***u*-GIIm**	***s*-GIIm**
C_6D_6	18.77	18.42	40.8	38.2
C_7D_8	18.65	18.29	40.7	38.0
CD_2Cl_2	18.09	17.73	39.9	37.1
$CDCl_3$	18.15	17.78	39.2	36.2
THF-d_8	18.21	17.86	40.3	37.6
R = Ph	***u*-GII**	***s*-GII**	***u*-GII**	***s*-GII**
C_6D_6	19.94	19.65	32.0	30.1
C_7D_8	19.84	19.55	32.0	30.0
CD_2Cl_2	19.38	19.08	32.1	29.9
$CDCl_3$	19.43	19.13	31.8	29.6
THF-d_8	19.55	19.25	31.9	29.9

[a] 300.1 MHz (^{1}H NMR) or 121.6 MHz (^{31}P{^{1}H} NMR); 22 °C.

7. Crystallographic details for *u*-GIIm.

Table S2. Crystallographic Details for ***u*-GIIm**.

A. Crystal data	
formula	$C_{40}H_{59}Cl_2N_2PRu$
formula weight	770.83
crystal dimensions (mm)	0.16 × 0.14 × 0.02
crystal system	monoclinic
space group	$P2_1/n$ (an alternate setting of $P2_1/c$ [No. 14])
unit cell parameters[a]	
a (Å)	12.2117 (3)
b (Å)	18.0562 (6)
c (Å)	18.2926 (6)
β (deg)	103.741 (2)
V (Å^3)	3918.0 (2)
Z	4
ρ_{calcd} (g cm^{-3})	1.307
μ (mm^{-1})	5.088
B. Data collection and refinement	
Diffractometer	Bruker D8/APEX II CCD[b]
radiation (λ [Å])	Cu Kα (1.54178) (microfocus source)
temperature (°C)	–100
scan type	ω and ϕ scans (1.0°) (5 s exposures)
data collection 2θ limit (deg)	135.98
total data collected	25622 ($-14 \le h \le 14$, $-21 \le k \le 21$, $-21 \le l \le 21$)
independent reflections	6912 (R_{int} = 0.1444)
number of observed reflections (*NO*)	4015 [$F_o^2 \ge 2\sigma(F_o^2)$]
structure solution method	Patterson/structure expansion (*DIRDIF–2008*)[1]
refinement method	full-matrix least-squares on F^2 (*SHELXL–97*)[2]
absorption correction method	Gaussian integration (face-indexed)
range of transmission factors	0.8876–0.4910
data/restraints/parameters	6912 / 1[c] / 429
extinction coefficient (x)[d]	0.00016(3)
goodness-of-fit (S)[e] [all data]	0.954
final R indices[f]	
R_1 [$F_o^2 \ge 2\sigma(F_o^2)$]	0.0517
wR_2 [all data]	0.0517
largest difference peak and hole	0.499 and –0.699 e Å^{-3}

[a]Obtained from least-squares refinement of 3223 reflections with 7.92° < 2θ < 105.86°.

[b]Programs for diffractometer operation, data collection, data reduction and absorption correction were those supplied by Bruker.

[c]The C1–H1A and C1–H1B distances were constrained to be equal (within 0.06 Å) during refinement.

[d]$F_c^* = kF_c[1 + x\{0.001F_c^2\lambda^3/\sin(2\theta)\}]^{-1/4}$ where k is the overall scale factor.

[e]$S = [\Sigma w(F_o^2 - F_c^2)^2/(n - p)]^{1/2}$ (n = number of data; p = number of parameters varied; $w = [\sigma^2(F_o^2) + (0.0490P)^2]^{-1}$ where $P = [\mathrm{Max}(F_o^2, 0) + 2F_c^2]/3$).

[f]$R_1 = \Sigma||F_o| - |F_c||/\Sigma|F_o|$; $wR_2 = [\Sigma w(F_o^2 - F_c^2)^2/\Sigma w(F_o^4)]^{1/2}$.

8. References

(1) Beurskens, P. T.; Beurskens, G.; de Gelder, R.; Smits, J. M. M.; Garcia-Granda, S.; Gould, R. O. *The DIRDIF-2008 Program System*, Crystallography Laboratory, Radboud University: Nijmegen, The Netherlands, 2008.

(2) Sheldrick, G. M. *Acta Crystallogr.* **2008**, *A64*, 112–122.
